# Tumor-promoting macrophages prevail in malignant ascites of advanced gastric cancer

**DOI:** 10.1038/s12276-020-00538-y

**Published:** 2020-12-04

**Authors:** Hye Hyeon Eum, Minsuk Kwon, Daeun Ryu, Areum Jo, Woosung Chung, Nayoung Kim, Yourae Hong, Dae-Soon Son, Seung Tae Kim, Jeeyun Lee, Hae-Ock Lee, Woong-Yang Park

**Affiliations:** 1 Samsung Genome Institute, Samsung Medical Center, Seoul, South Korea; 2 Division of Hematology-Oncology, Department of Medicine, Samsung Medical Center, Sungkyunkwan University School of Medicine, Seoul, Korea; 3 Department of Health Sciences and Technology, Samsung Advanced Institute for Health Sciences & Technology, Sungkyunkwan University, Seoul, South Korea; 4 School of Big Data Science, Data Science Convergence Research Center, Hallym University, Chuncheon, South Korea; 5 Department of Biomedicine and Health Sciences, Graduate School of The Catholic University of Korea, Seoul, South Korea; 6 Department of Molecular Cell Biology, Sungkyunkwan University School of Medicine, Suwon, South Korea

**Keywords:** Gastric cancer, RNA sequencing, Cancer genomics

## Abstract

Gastric cancer (GC) patients develop malignant ascites as the disease progresses owing to peritoneal metastasis. GC patients with malignant ascites have a rapidly deteriorating clinical course with short survival following the onset of malignant ascites. Better optimized treatment strategies for this subset of patients are needed. To define the cellular characteristics of malignant ascites of GC, we used single-cell RNA sequencing to characterize tumor cells and tumor-associated macrophages (TAMs) from four samples of malignant ascites and one sample of cerebrospinal fluid. Reference transcriptomes for M1 and M2 macrophages were generated by in vitro differentiation of healthy blood-derived monocytes and applied to assess the inflammatory properties of TAMs. We analyzed 180 cells, including tumor cells, macrophages, and mesothelial cells. Dynamic exchange of tumor-promoting signals, including the CCL3–CCR1 or IL1B–IL1R2 interactions, suggests macrophage recruitment and anti-inflammatory tuning by tumor cells. By comparing these data with reference transcriptomes for M1-type and M2-type macrophages, we found noninflammatory characteristics in macrophages recovered from the malignant ascites of GC. Using public datasets, we demonstrated that the single-cell transcriptome-driven M2-specific signature was associated with poor prognosis in GC. Our data indicate that the anti-inflammatory characteristics of TAMs are controlled by tumor cells and present implications for treatment strategies for GC patients in which combination treatment targeting cancer cells and macrophages may have a reciprocal synergistic effect.

## Gastric cancer: Understanding cellular interactions suggests potential treatments

New strategies for treating advanced gastric cancer could emerge from insights into the interactions between white blood cells called macrophages and tumor cells in fluid known as malignant ascites that accumulates in the abdomen. Researchers in Seoul, South Korea, led by Hae-Ock Lee at The Catholic University of Korea and Woong-Yang Park at the Samsung Medical Center compared macrophages from healthy subjects with those from gastric cancer ascites. They identified molecular signaling interactions between tumor cells and macrophages that recruited macrophages into the ascites and converted them into more anti-inflammatory forms. The macrophages were then able to promote the activities of the cancer cells. The results suggest that chemicals able to inhibit or deplete proteins now identified as involved in controlling these synergistic interactions could become a new class of therapeutic agents.

## Introduction

Gastric cancer (GC) is a highly heterogeneous disease in histopathological, molecular and clinical aspects. To uncover the heterogeneity and to broaden the molecular understanding of cancers, comprehensive genomic characterizations have been performed throughout various cancers. The Cancer Genome Atlas (TCGA) analysis reported four molecular subtypes of GC: chromosomal instability (CIN), microsatellite instability-high (MSI), genomically stable (GS), and Epstein-Barr virus (EBV)^1^. However, the clinical significance of these subtypes needs to be elucidated.

The Asian Cancer Research Group (ACRG) reported four GC molecular subtypes that are associated with recurrence pattern and prognosis: microsatellite-stable (MSS)/TP53−, MSS/TP53+, MSI, and MSS/epithelial-to-mesenchymal transition (EMT). GC patients with the MSS/EMT subtype showed significantly more frequent recurrence, a higher rate of peritoneal carcinomatosis (PC) at recurrence, a younger age at presentation, and poorer survival than patients in other subtypes^2^. The response to systemic chemotherapy in GC patients with PC is poor, and the presence of PC is an independent poor prognostic factor in advanced GC (AGC)^3^.

While immune checkpoint blockade showed excellent clinical response in MSI and EBV subtypes, GC patients with the EMT subtype did not benefit from immune checkpoint blockade^4,5^. We reported that GC patients with the mesenchymal subtype showed a poor response to pembrolizumab despite an increased immune signature^4^. EMT mediates resistance to immunotherapies by recruiting immunosuppressive macrophages and neutrophils (M2 macrophages and myeloid-derived suppressor cells) such that blocking the immune checkpoint axis is not sufficient to restore antitumor immunity^6^.

Recent studies investigating the tumor microenvironment with single-cell RNA sequencing (scRNA-seq) technology identified that macrophages in tumors exhibited a distinct state or spectrum, which is more complex than the classical binary M1/M2 model^7–9^. Comprehensive intercellular interactions between tumor cells and immune cells were investigated by analyzing receptor and ligand expression through scRNA-seq^10^. However, few studies have characterized the immunosuppressive niche in the ascites of GC patients.

In the current study, we used scRNA-seq to explore heterogeneity and functional interactions in both immune cells and tumor cells in ascites of GC patients. Using the microfluidic C1 system, we captured tumor cells and macrophages in malignant ascites. Tumor-specific gene expression profiling revealed patient-specific as well as heterogeneous gene expression signatures for stemness, EMT, and molecular-targeted therapies. Most macrophages manifested an alternatively activated ‘M2’ phenotype, which seems to be influenced by the metastatic sites as well as associated tumor types. Overall, our data demonstrate the aggressive characteristics of peritoneal GC cells in terms of their potential for stimulating growth and shaping a tumorigenic microenvironment.

## Materials and methods

### Preparation of cells from patient-derived specimens

All patients and healthy donors in this study agreed to provide biospecimens through a consent form approved by the Institutional Review Board of Samsung Medical Center (ClinicalTrials.gov. Identifier: NCT#02299648^11^ and Institutional Review Board nos. 2016-04-107 and 2017-04-038).

Five samples were collected from four patients diagnosed with metastatic GC. We obtained four peritoneal ascites samples and one cerebrospinal fluid (AGC04CSF) sample aspirated for treatment purposes. Normal peritoneal cells were collected from peritoneal dialysates of three donors free of cancer, peritonitis, bacterial infection, and hepatitis B/C virus. Suspended cells were collected by centrifugation, and cell debris was removed by Ficoll-Paque^TM^ PLUS (GE Healthcare, Uppsala, Sweden) separation.

### In vitro differentiation of M1-type or M2-type macrophages

Fresh PBMCs from two healthy donors were separated from whole blood samples using Ficoll-Paque^TM^. CD14+ monocytes were selected using MACS human CD14 MicroBeads (Miltenyi Biotec GmbH, Bergisch Gladbach, Germany), pre-separation filters (Miltenyi), and LS columns (Miltenyi) following the manufacturer’s recommendations. To induce M0 macrophages, isolated CD14+ monocytes were seeded onto FBS-coated 24-well plates at a density of 1 × 10^5^ cells/cm^2^. Seeded cells were cultured for 7 days in RPMI 1640 media supplemented with 20% FBS and 100 ng/ml M-CSF (BioLegend, San Diego, CA, USA). On day 7, M-CSF-containing medium was removed, and appropriate stimulating media containing 100 ng/ml LPS (Sigma-Aldrich, St. Louis, MO, USA) and 20 ng/ml IFN-γ (BioLegend) for M1 induction or 20 ng/ml IL-4 (BioLegend) and 20 ng/ml IL-10 (BioLegend) for M2 induction were supplied. After an additional 48 h of culture, cells were collected by gentle scraping and then taken for fluorescence-activated cell sorting (FACS) analysis or scRNA-seq. The morphological changes that occurred during differentiation were observed every 2 days under a microscope. A fraction of the cells was triple-stained with PE/Cy7 anti-human CD14 antibody (BioLegend), FITC anti-human CD80 antibody (BioLegend), and PE anti-human CD163 antibody (BioLegend) and analyzed by FACSVerse and FACSuite v1.2 (BD Biosciences).

### Full-length single-cell RNA sequencing and data processing

Single-cell transcriptome data for GC, M1/M2 macrophages, and normal peritoneal cells were prepared using the C1^TM^ Single-Cell AutoPrep System (Fluidigm, San Francisco, CA, USA) following the manufacturer’s instructions. Freshly prepared cell suspensions were loaded onto the C1 system, and amplified cDNAs were obtained. The type of microfluidic chip used in the C1 system was determined by the size distribution and average cell size of each sample. We quantified the amount of amplified cDNA using a Qubit^®^ 2.0 Fluorometer (Life Technologies, Carlsbad, CA, USA) and then assessed the quality using 2100 Bioanalyzer (Agilent Technologies, Santa Clara, CA, USA). Successfully amplified cDNA from single cells (180 GC cells, 97 M1 cells, 45 M2 cells, and 25 normal peritoneal cells) were subjected to RNA sequencing.

Sequencing libraries were constructed with the Nextera XT DNA Sample Prep Kit (Illumina) and sequenced with the HiSeq 2500 system in 100-bp paired-end mode using the TruSeq Rapid PE Cluster kit and the TruSeq Rapid SBS kit. The sequence reads were aligned to the UCSC hg19 human reference genome using the two-pass mode of STAR_2.4.0b (default parameters)^12^, and the transcript per million (TPM) value of each gene was quantified using RSEM v1.2.17 with default parameters^13^. The TPM_*ij*_ value for gene *I* in cell *j* was derived by summing the TPM values of the isoforms of gene *i*. To reduce the inflation effect of the TPM calculations on gene expression levels (Es), we defined *E*_*ij*_ = log_2_(TPM_*ij*_/10 + 1) as the gene expression value, as described previously^14^.

Unreliable cells expressing fewer than 1000 genes and unreliable genes expressed in fewer than 10 cells were excluded from further analysis. When filtering cells or genes, *E*_*ij*_ > 1 was considered confident expression. Finally, 162 cells from GC patients, 97 M1 cells, 45 M2 cells, and 25 cells from normal peritoneum (peritoneal dialysate) were used. For normal peritoneal data, we counted unreliable genes as being expressed in fewer than 2 cells instead of 10 due to the small sample size. Based on marker gene expression, nine of the 25 normal peritoneal cells were considered macrophages (*E*_*j*_ for CD86 > 2, MUC16 < 1.1, EPCAM = 0, and CD3D = 0) and used for further analysis.

### Batch correction using Harmony

Batch correction was performed using the RunHarmony function of the R package ‘harmony (v1.0)’^15^ (Supplementary Fig. 1c). To compare the effect of batch correction on cell type identification, the Jaccard index was calculated using the ‘jaccard’ function of the R package ‘jaccard (v0.1.0)’, and Fisher’s exact test was performed using the ‘fisher.test’ function of the R package ‘stat’.

### Droplet-based single-cell RNA sequencing and data processing

Massively parallel droplet-based data from malignant ascites of AGC04 patient was generated to target 5000 cells using the Chromium System (10× Genomics, Pleasanton, CA, USA) with the Single Cell 3′ Reagent Kit (v2) following the manufacturer’s instructions. Libraries were sequenced on the HiSeq 2500 system, and reads were aligned to the GRCh38 human reference genome using Cell Ranger v2.1 software with default options.

Expression matrix processing, cell clustering, and dimensionality reduction were performed using Seurat v3.0. The gene Es for gene *i* in cell *j* was defined as *E*_*ij*_ = log_2_(pseudoTPM_*ij*_ + 1), where pseudoTPM_i*j*_ = UMI_*ij*_/sum[UMI_total*,j*_] * 10,000. Cells were clustered by the graph-based shared nearest-neighbor (SNN) method, and cells in cluster 0 and 4 that exhibited differentially expressing macrophage markers (such as CD68) were defined as macrophages. Low-quality cells that did not meet any of the following criteria were excluded from the macrophage analysis: (1) 1000 ≤ nCounts ≤ 150,000, (2) 200 ≤ nFeatures ≤ 10,000, and (3) percent of mitochondrial genes ≤ 20.

### Processing of public dataset

Full-length data for breast cancer^16^ and colorectal cancer^17^ were processed as described above using raw fastq files, and then the macrophages identified in the original study were used after removing unreliable cells. Cell clustering and dimensionality reduction analysis were performed using Seurat v3.0^18^. Public 10× data for primary GC^19^ and antral mucosa biopsies^20^ were subjected to data processing and unreliable cell removal as described in the original study, and macrophage clusters were identified based on marker gene expression. For the ovarian cancer ascites dataset^21^, the macrophages identified in the original study were used.

### Chromosomal expression pattern (CEP) of single-cell RNA-seq data

The CEP reflecting copy number variation (CNV)^14,22,23^ was estimated from single-cell RNA-seq data. First, we performed *Z*-normalization on the expression values of autosomal genes and limited the *Z*-score range to [−3, 3]. After sorting the genes by their genomic positions (from chromosome 1 to chromosome 22), moving average values were calculated with a sliding window of 150 genes within each chromosome and then adjusted by centering. To estimate the CNV instability in each cell, we calculated the mean of squares (MS) of the CEP values. The mean difference in the MS values between epithelial cells and macrophages was determined by *t*-test, and mesothelial cells were excluded from statistical analysis because there were too few of this group (*n* = 5).

### Cell–cell interaction analysis

Based on the Es of ligand–receptor pairs listed in the FANTOM5 database^24^, cellular interactions were inferred by assuming crosstalk between cells expressing the ligand and cells expressing the counterpart receptor. The ligand–receptor interaction was inferred from the binary expression of the ligand or receptor genes, with an expression value (*E*_*ij*_) threshold of 1. If one cell expresses ligand (*E*_*ij*_ > 1) and another cell expresses the counterpart receptor (*E*_*ij*_ > 1), we counted this ‘expression pair’ as the ‘intercellular interaction’. If one cell expressed both ligand and receptor genes (*E*_*ij*_ > 1), we counted this expression pair as ‘self-sufficient signaling’. Additional ligand–receptor pairs for cytokine/chemokine signaling were collected from previous studies (listed in Supplementary Table 1).

### Extraction of M1/M2-specific signatures

The M1-specific or M2-specific signature gene sets were constructed afresh using robust genes at single-cell resolution. First, we compared M1 and M2 cells in each donor. Expected counts from the RSEM of M1 and M2 cells were compared by LRT using edgeR^25^ and DESeq2^26^. Both edgeR and DESeq2 recommend expected counts as input and provide LRT functions to extract differentially expressed genes from single-cell data. Normalization by the trimmed mean of the *M*-values (TMM) was performed before the LRT test when using edgeR. Thereafter, we filtered for markers for M1 and M2 macrophages. Genes with logFC > 2 (edgeR) or log2FoldChange > 2 (DESeq2) and FDR < 0.05 were obtained from each method. Finally, common markers from the two methods (Supplementary Fig. 4c) and from the two donors were selected. A total of 155 and 43 genes configured M1 and M2 signature gene sets, respectively.

### Pathway analysis

**Fig. 1:**
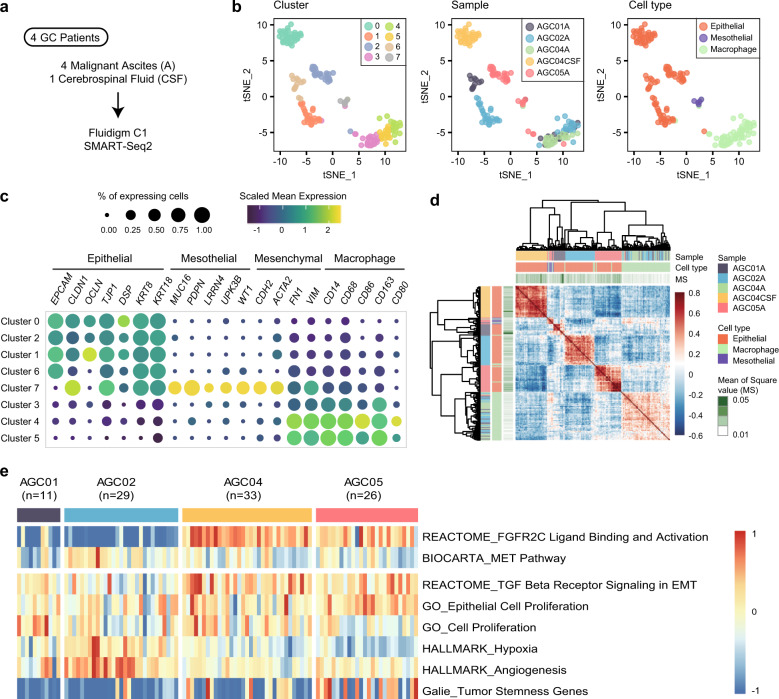
Characterization of tumor cells and tumor-associated macrophages in malignant ascites. **a** Scheme of data generation in the study. **b** Dimension reduction using t-distributed stochastic neighbor embedding (tSNE) separates noncancerous cell clusters and patient-specific cancer cell clusters. Each dot represents a cell, and each cell is colored by its SNN cluster (left), sample origin (middle), or cell type (right). **c** The expression of marker genes for epithelial cells, peritoneal mesothelial cells, mesenchymal cells, and macrophages clarified the cell type of the clusters. **d** Pearson’s correlation coefficient matrix of the CEP values. **e** Heatmap of tumor characteristics in the tumor cells. Each column represents a single cell.

To assess gene expression signatures and pathway activation, the signature Es was evaluated as *E*_*sj*_(*S*) = average [*E*_1*…n, j*_], where *S* is a gene set consisting of *n* genes. Public gene sets used for pathway analyses were collected from either the Molecular Signature Database (MSigDB) v6.1^27^ or previous research papers (bulk-derived M1/M2 gene sets^28^ and curated M1/M2 gene sets^7^). The signature Es for gene set *S* was rescaled to [−1, 1] in Fig. 1e. The simple linear regression model was created by the ‘lm’ function of the R package ‘stat’ to show inclination toward the M2 axis (Fig. 4 and Supplementary Figs. 5–7).

### Survival analyses with the macrophage-specific gene signature

For the survival analysis, the expression data (RNA sequencing, level 3) of stomach adenocarcinoma (STAD), colorectal adenocarcinoma (COAD), and breast invasive carcinoma (BRCA) datasets were obtained from TCGA (updated in 2017). Only samples with clinical information on pathological stage and survival information were used for further analysis (STAD, *n* = 378; COAD, *n* = 270; BRCA, *n* = 1059). The Es for gene *i* in sample *l* was defined as *E*_*il*_ = log_2_ (TPM_*il*_/10 + 1). For gene set *S* consisting of *n* genes, the signature Es was evaluated as *E*_sl_(*S*) = average[*E*_1*…n, l*_] only for the genes detected in the bulk RNA-seq data. The ‘high’ group and ‘low’ group for Kaplan–Meier analysis were determined by the 25th and 75th percentiles. Survival curves and significance were estimated using the Kaplan–Meier formula of the R packages ‘survival’ and ‘survminer’.

## Results

### Identification of cell types in malignant ascites of GC

For cellular characterization of malignant ascites, we obtained full-length scRNA-seq data for 180 cells from four patients by the SMART-Seq2 method in the C1 microfluidic system (Table 1)^29^. For one patient, cerebrospinal fluid (CSF) metastasis was analyzed in parallel (Fig. 1a). Unsupervised clustering using a SNN algorithm was used for cell classification after principal component analysis (PCA), and the SNN graph was projected using t-distributed stochastic neighbor embedding (tSNE)^30^. Five patient-specific clusters (0, 1, 2, 6, and 7) and three multipatient clusters (3, 4, and 5) were classified (Fig. 1b and Supplementary Fig. 1a). From the literature review, we expected a collection of GC cells, tumor-associated immune cells, and peritoneum-derived mesothelial cells as the main cell types^31,32^. Indeed, marker genes indicated the cluster identities (Fig. 1b right); four (clusters 0, 1, 2, and 6) were epithelial cancer cells, one (cluster 7) was mesothelial cells, and three (clusters 3, 4, and 5) were macrophages (Fig. 1c and Supplementary Fig. 1b)^33–36^.

**Table 1:** Clinical information of specimen donors.

Patient	AGC01	AGC02	AGC04	AGC05
Diagnosis	Adenocarcinoma, poorly differentiated	Signet ring cell carcinoma	Adenocarcinoma, poorly differentiated	Signet ring cell carcinoma
Metastases	Ascites	Ascites	Ascites, cerebrospinal fluid (CSF)	Ascites
Stage	IV	IV	IV	IV
Age	51	78	45	42
Sex	Male	Male	Female	Female
MSI type	MSS	n/c	MSS	MSS
Genomic aberrations	PIC3CA mutation	–	FGFR2 amplification	FGFR2 amplification

Considering the potential batch effect derived from samples, the cell types identified with or without sample batch correction were compared. The odds ratio was 140.81 for epithelial cells, 77.61 for mesothelial cells, and 393.24 for macrophages (*p*-value < 0.001), suggesting no significant batch effect in our data analysis (Supplementary Fig. 1c). In ascites of AGC04 (AGC04A), we failed to find any epithelial cells. To investigate whether the absence of epithelial cells in AGC04A is real or sampling bias, we generated large-scale data for AGC04A via a massively parallel droplet-based single-cell RNA-seq method and analyzed the transcriptomes of 1768 cells. As a result, we recovered a small number of epithelial tumor cells along with large numbers of macrophages and T cells (Supplementary Fig. 2). These results demonstrate the limitation of the C1 platform in capturing the comprehensive cellular landscape of the tumor microenvironment. Therefore, we focused on the specific molecular features of two major populations in GC ascites in the downstream analysis: tumor cells and macrophages.

Most cells from cluster 7 expressed both epithelial and mesenchymal markers, including *KRT8*, *KRT18*, *CLDN1*, *TJP1*, *CDH2*, and *ACTA2*. These gene expression features suggest EMT of mesothelial cells and consequent damage to the peritoneal membrane when GC spreads to the peritoneum^37^. Mesothelial cells, despite their small numbers, were separately clustered from the epithelial cells in the global (Fig. 1b and Supplementary Fig. 2a) and CEPs (Supplementary Fig. 3a). We used CEP to infer CNVs as a hallmark of malignant cells. The epithelial cell clusters demonstrated patient-specific CEP, with higher copy number fluctuations than the mesothelial cell or macrophage cell clusters (Supplementary Fig. 3b). Hierarchical clustering using Pearson’s correlation coefficient of CEPs distinguished four epithelial tumor groups, one mesothelial group, and one macrophage group, thus recapitulating the clusters in global gene expression (Fig. 1d). The four epithelial tumor clusters showed patient-specific tumor characteristics (high FGFR2 or MET) and intratumoral heterogeneity in gene expression signatures of cancer-related processes, such as cell proliferation, stemness, TGF-β signaling in EMT, hypoxia, and angiogenesis (Fig. 1e).

### Interaction between tumor cells and macrophages in malignant ascites

**Fig. 2:**
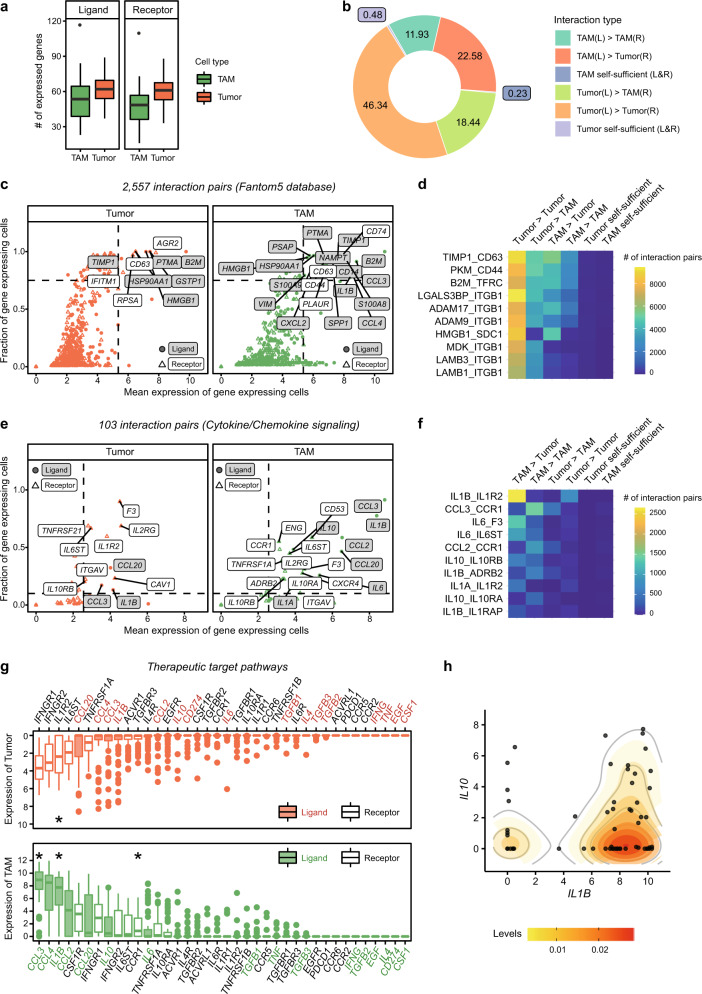
Tumor-promoting interactions of cancer cells and macrophages in malignant ascites. **a** The number of expressed ligand (left) or receptor (right) genes from the FANTOM5 database in each cell. **b** Putative intercellular interactions between two other cells are much more prominent than was putative self-sufficient signaling. Intercellular interaction types were indicated by ‘ligand-expressing cell (L)>receptor-expressing cell (R)’. **c** Strongly and commonly expressed interacting genes in tumors (left) or TAMs (right). The genes with average expression over quantile 0.95 and a fraction of expressing cells over 0.75 were labeled. **d** Top 10 abundant interaction pairs (>10,224 pairs in each interaction) related to **c**. **e** Strongly and commonly expressed interacting genes among the chemokine-related interactions (Supplementary Table 1a). Genes with average expression over quantile 0.5 and a fraction of expressing cells over 0.1 were labeled. **f** Top 10 abundant interaction pairs (>406 pairs in each interaction) related to **e**. **g** Expression level of the gen**e**s associated with macrophage function (Supplementary Table 1b) indicates high expression of *CCL3*, *IL1B*, and *CCR1* in macrophages and *IL1R2* in tumor cells (marked with a star). **h** Coexpression of *IL1B* and *IL10* in TAMs. The *x* and *y*-axes show the expression levels of *IL1B* and *IL10*, respectively. Each dot represents a cell, and each cell was colored based on the sample origin. Expressed genes were evaluated based on threshold 1 in **a–f**.

The concerted action of the tumor and the associated microenvironment generates a unique ecosystem promoting tumor growth and invasion in a metastatic setting. To delineate the cellular interplay in malignant ascites, we inferred the molecular interactions by quantifying the expression of ligand–receptor pairs listed in the FANTOM5 database^24^. When we enumerated the expression of ligands and receptors in tumor cells and macrophages (Fig. 2a), the paired expression trended more toward intercellular interactions than self-sufficient signaling (Fig. 2b). The most common interaction pairs were composed of adhesion molecules with a wide range of specificity (Fig. 2c and d). Among them, tissue inhibitor of metalloproteinase 1 (TIMP1) can promote cell proliferation^38^ and protect cells from hypoxia-induced apoptosis via TIMP1-CD63 signaling^39^. Integrin beta 1 subunit (ITGB1), together with multiple integrin alpha subunits, can interact with pleiotropic ligands and promote cancer cell proliferation and metastasis^40,41^.

Next, we focused on more specific interactions that mediate cytokine or chemokine signaling (Supplementary Table 1)^42,43^. The majority of tumor-associated macrophages (TAMs) expressed proinflammatory cytokine/chemokine genes, such as *IL1B*, *CCL2*, *CCL3*, and *CCL20* (Fig. 2e). Interestingly, the most abundant cytokine–receptor pair interaction between TAMs and tumor cells was predicted for *IL1B* and its decoy receptor *IL1R2* (Fig. 2f and g), suggesting the inhibition of IL1B-mediated proinflammatory signaling by tumor cells. In addition, the IL10–IL10RA interaction within TAM populations also limits inflammatory immune responses. Indeed, many TAMs coexpress *IL1B* and *IL10* (Fig. 2h). The CCL2/CCL3–CCR1 interaction within TAM populations may represent a distal loop for macrophage recruitment (Fig. 2f and g). Altogether, these results provide a systemic view of the molecular and cellular network in malignant ascites, fostering an anti-inflammatory microenvironment and supporting tumor growth and invasion.

### Construction of the reference macrophage transcriptome at single-cell resolution

**Fig. 3:**
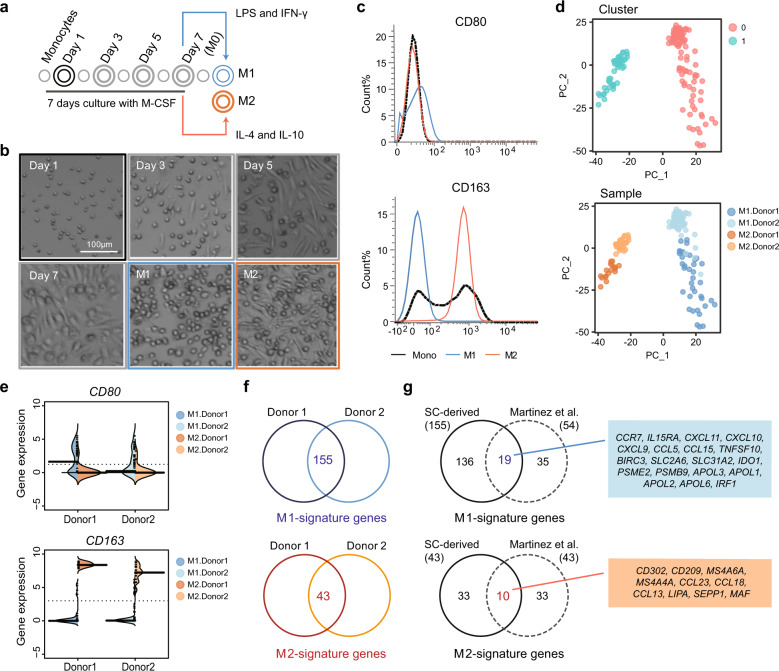
M1- and M2-signature genes at single-cell resolution. **a** Scheme of the in vitro differentiation of M1-type and M2-type macrophages. **b** Morphological changes during differentiation. **c** FACS analysis of the M1-specific marker CD80 and the M2-specific marker CD163 in differentiated M1 and M2 cells. The examined samples are colored with borders in **a** (black for monocytes, blue for M1 macrophages, and orange for M2 macrophages). **d** Unsupervised PCA primarily separated M1 and M2 macrophages by the first principal component. Cells are colored by cluster (upper) or sample origin (lower). **e** Gene expression levels of *CD80* and *CD163* in single-cell RNA-seq data confirmed M1 and M2 polarization. The thick black line indicates the median value of each sample. **f** M1-specific or M2-specific signature genes were extracted by overlapping differentially expressed genes from both donors. **g** Comparison with published gene sets derived from bulk M1 and M2 signatures^28^. Only 29 genes were repeatedly extracted from single-cell and bulk signatures.

In different tissues and disease conditions, macrophages show a broad phenotypic spectrum in their inflammatory nature, ranging from the proinflammatory M1 type to the anti-inflammatory M2 type. Although studies have shown that macrophages have various states beyond the dichotomous definition of M1 and M2, extremely differentiated proinflammatory M1 and anti-inflammatory M2 macrophages are still valuable as reference points for determining the characteristics of macrophages. M2-like macrophages show tumor-promoting activity in vitro, and a high M2/M1 macrophage ratio is associated with poor prognosis in GC^44^. To provide quantifiable gene expression criteria for M1 or M2 characteristics, we generated a single-cell transcriptome reference for these macrophage populations (Fig. 3a). Briefly, we isolated monocytes from the blood of two healthy donors, cultured them with M-CSF, and then induced differentiation to the M1 or M2 type with LPS/IFN-gamma or IL-4/IL-10, respectively. Cellular morphology and surface marker expression confirmed successful induction of M1 and M2 macrophages (Fig. 3b, c). Finally, 97 M1 and 45 M2 cells were captured and subjected to full-length mRNA sequencing in the C1 platform. PCA demonstrated that M1-type and M2-type macrophages have highly variable transcription features and can be separated by the first principal component (Fig. 3d, Supplementary Fig. 4a and b). The second principal component separated the two donors, indicating donor-specific gene expression variability (Fig. 3d). However, differential gene expression pattern between the M1-type and M2-type macrophages was similar for the two donors (Fig. 3e).

To extract differentially expressed genes from M1 and M2 macrophages, we used the likelihood ratio test (LRT) on zero-inflated data for each donor (Fig. 3f, Supplementary Fig. 4c, and Supplementary Table 2). A total of 155 and 43 genes were concurrently extracted from two donors as M1-specific and M2-specific signatures, respectively. Among these, 29 genes overlapped with the M1-specific or M2-specific gene sets generated from pooled cells using microarray data (Fig. 3g)^28^. These 29 overlapping genes showed high levels of differential expression between M1 and M2 (Student’s *t*-test *p-*value < 1e5) (Supplementary Fig. 4d) and may be used as platform-independent gene expression markers for distinguishing the two macrophage types at both bulk and single-cell levels.

### M1–M2 polarization map of TAMs

**Fig. 4:**
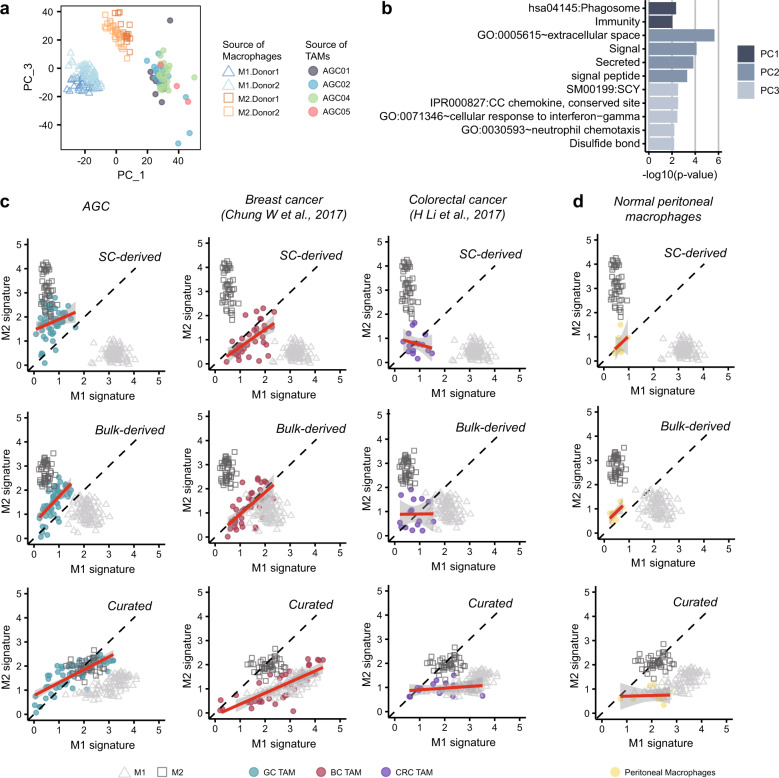
In vitro differentiated M1 and M2 transcriptomes show the M1-like and M2-like features of TAMs. **a** PCA grouped TAMs into a separate cluster from the reference M1 or M2 cells. Cells were colored based on the origin of the sample. **b** The GO terms describing each principal component show the functional difference between macrophage types (Bonferroni corrected *p*-value < 0.01). The top 50 genes on each principal component were analyzed by DAVID 6.8^53^. **c** Two-dimensional dot plots for M1 and M2 signature evaluation were constructed for three cancer types using three different gene sets: single-cell transcriptome-derived signatures (top), bulk transcriptome-derived signatures (middle), and curated signatures (bottom). The *x*-axis indicates expression in the M1 signature, and the *y*-axis indicates expression in the M2 signature. The red line represents the simple linear regression line, and the gray area represents the confidence interval. **d** The M1–M2 signature 2-D plot estimates the M1-like and M2-like phenotypes of normal peritoneal macrophages.

Using the single-cell transcriptome as a reference, we assessed the M1/M2 polarization status of TAMs in malignant ascites of GC. PCA distinguished global transcriptomic profiles of TAMs from M1 or M2 reference cells, which is consistent with previous findings that distinctive macrophage states exist in tumors (Fig. 4a)^7–9^. Gene ontology analysis for each PC suggests that TAMs differ from M1 or M2 macrophages in phagocytic activity but are similar to M1 macrophages in chemokine secretion and response to external stimuli such as IFN-γ (Fig. 4b). Consistently, we have shown via cellular interaction analysis that TAMs abundantly express proinflammatory cytokines (Fig. 2). However, the M1 versus M2 polarization map suggests that TAMs from GC ascites are more like M2 macrophages than M1 macrophages. In a comparative analysis of M1 vs. M2 polarization, our single cell-derived signatures separated the reference cells well and positioned the GC ascites TAMs toward the M2 axis (Fig. 4c, top left). These separation patterns were similarly repeated in the bulk microarray-derived signatures (Fig. 4c middle left)^28^. The curated signatures (Fig. 4c bottom left)^7^ failed to position reference cells in the M2 axis but endowed similar polarization status for the M2 reference cells and GC ascites TAMs. Thus, all three methods ascertained that TAMs from the malignant ascites of GC resemble M2 reference cells more than M1 reference cells. The 10× data for AGC04A consistently show the M2-like characteristics and patterns of GC ascites TAMs when accounting for all three gene sets (Supplementary Fig. 5). Application of M1 vs. M2 scoring of TAMs from breast or colorectal cancer tissues demonstrated less inclination toward the M2 axis, indicating a strong M2-like polarization status of GC ascites TAMs (Fig. 4c).

The M2 polarization states for different TAMs might have been influenced by tissue origin and the cell isolation process. Some studies have shown that peritoneal macrophages have an anti-inflammatory M2 phenotype^45^, and enzymatic dissociation of solid tumor tissues may induce inflammatory gene expression^46^. To address the first issue, we compared the M1 and M2 scores in normal peritoneal macrophages (Fig. 4d). Macrophages released from the normal peritoneum show a slight M2-like phenotype but a nearly balanced polarization state, indicating that the strong M2 polarization of GC macrophages is not the sole representation of tissue origin. In addition, the TAMs of primary GC show only a modest inclination toward M2 macrophages in the curated gene set, while there were no inclinations for normal tissues, peripheral blood, or early GC^19^ (Supplementary Fig. 6a). The independent data repeatedly show a balanced state of macrophages in nontumor stomach or early GC^20^ (Supplementary Fig. 6b). In malignant ascites of ovarian cancer^21^, TAMs show a pattern trending toward M2 (Supplementary Fig. 7). We addressed the second issue by comparing a list of dissociation-induced gene (such as *EGR1, FOS, FOSB, JUN, JUNB, SOCS3*, *HSP90AA1*, *HSPA1A*, *HSPA1B*, and *HSPA8*)^46^ and our M1/M2-specific genes. None of these genes were included in the M1 or M2 signature gene lists.

These data suggest that the experimentally derived single cell-level gene sets provide an objective reference for macrophage polarization and that using a reference transcriptome would prevent incorrect conclusions from being made due to internal comparison.

### Prediction of overall survival using M2-specific signature genes in GC

**Fig. 5:**
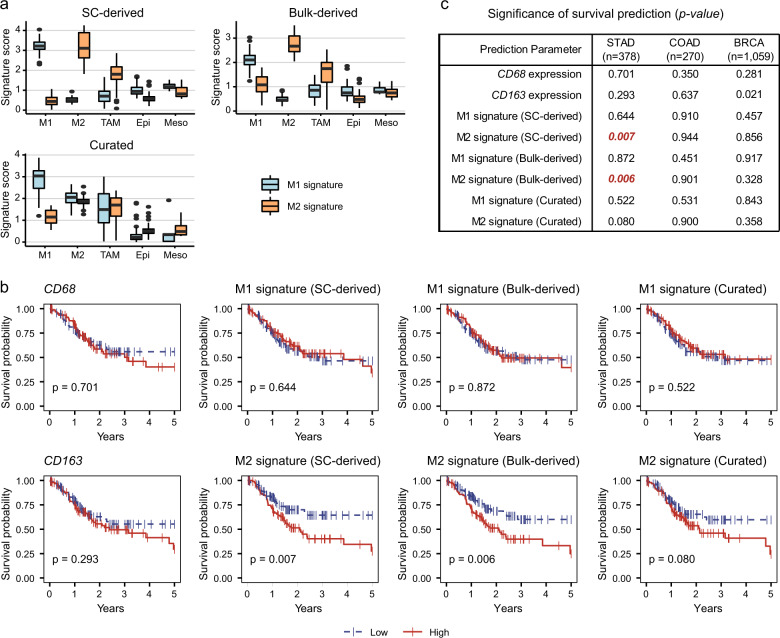
The M2 phenotype of TAMs can be applied to predict the prognosis in gastric cancer. **a** Transcriptome-driven M2 signatures, not curated signatures, are high exclusively in M2-type cells. M1 reference M1 cells, M2 reference M2 cells, TAM macrophages from GC, Epi epithelial cells from GC, Meso mesothelial cells from GC. **b** Overall survival prediction was performed using the TCGA STAD dataset (*n* = 378) and either signature gene sets or canonical macrophage markers, such as *CD68* and *CD163*. The ‘high’ group (red) and ‘low’ group (blue) were determined by the 25th and 75th percentiles, respectively. *p* indicates the *p*-value of log-rank test. **c** Summary table of *p*-values for overall survival using the TCGA dataset for the three cancer types (STAD, *n* = 378; COAD, *n* = 270; and BRCA, *n* = 1059).

When characterizing TAMs from the bulk tissue expression data, non-macrophage cells expressing macrophage signature genes may interfere with TAM profiling. To test the exclusive expression of macrophage signature genes, we compared single-cell transcriptome profiles of M1 and M2 reference cells with those of TAMs, epithelial cells, and mesothelial cells from malignant ascites of GC (Figs. 1 and 5a). In the evaluation of M1 and M2 reference cells, our single-cell-derived or bulk microarray-derived gene sets showed good performance in the distinction of reference cells. Importantly, both M2 signatures maintained exclusivity in TAMs, showing much higher expression than that in epithelial and mesothelial cells. However, the level of M1 signature gene expression was comparable or higher in the non-macrophage populations. These results imply that the single-cell-derived and bulk microarray-derived M2 signature can be used to evaluate TAMs in bulk tissue data, whereas the M1 signature is not appropriate for evaluating the characteristics of macrophages in bulk tissue data. By comparison, the curated gene sets and M2 signature in particular showed a poor performance in representing reference cells.

To investigate the prognostic value of macrophage-specific signatures, we performed survival analysis on the TCGA bulk RNA-seq dataset (STAD, *n* = 378) (Fig. 5b). GC patients with a ‘high’ M2 signature had considerably worse survival than did the patients with a ‘low’ M2 signature (*p*-value 0.007 for the SC-derived M2 signature; 0.006 for the bulk-derived M2 signature). In the TCGA STAD set, the M1 signature and canonical macrophage markers, such as CD68 and CD163, showed no prognostic association (*p-*value > 0.05, Fig. 5b). As no M2 inclination was observed in breast cancer and colorectal cancer (Fig. 4c), we expected that the M2 signature could not predict survival in breast cancer and colorectal cancer. Indeed, none of the M1 or M2 signatures were associated with survival in COAD (*n* = 270) or BRCA (*n* = 1059) (Fig. 5c). Only CD163, not M2-signature, showed an association with poor prognosis in breast cancer (*p*-value = 0.021).

We further tested the prognostic value of individual genes comprising the M2-specific signature gene set to verify the effect of a single gene on the M2 signature and found that six genes (*CITED2*, *DACT1*, *CCDC152*, *DAB2*, *SEPP1*, and *THBD*) retained statistical significance for predicting survival (*p*-value < 0.05, Supplementary Fig. 8a–c). Nevertheless, five of these six genes had limited power in representing the TAM population in vivo. *DACT1* and *CCDC152* showed low expression in GC TAMs, and *DAB2*, *SEPP1*, and *THBD* were abundantly expressed in non-macrophage populations as well as in macrophages (Supplementary Fig. 8a and d). Gene expression in non-macrophage populations indicates contributions from these cells. By contrast, *CITED2* (CBP/p300-interacting transactivator with glutamic acid/aspartic acid-rich carboxyl-terminal domain 2) was expressed exclusively in TAMs or M2-type macrophages, suggesting a more specific role of this gene in the anti-inflammatory polarization of macrophages. Excluding each gene from the single-cell-derived M2 signature maintained a negative association with the survival rate (Supplementary Fig. 9). These results suggest that a single-cell-derived M2 signature can be used as a robust predictive parameter for survival.

Taken together, these data support the use of an M2-specific 43-gene expression signature for assessing the anti-inflammatory features of TAMs and predicting prognosis in GC with minimal interference from non-macrophage cells.

## Discussion

We showed that TAMs from malignant ascites in GC have strong M2-like characteristics and that this M2-like phenotype of TAMs is associated with poor prognosis in GC. Based on the intercellular interactions between tumor cells and macrophages, we speculated on the recruitment of macrophages and their transition to TAMs with anti-inflammatory properties. Since macrophages manifest a wide range of phenotypic and functional flexibility in vivo, we first dichotomized macrophage references as pro-inflammatory M1 or anti-inflammatory M2 cells and then systemically ordered TAMs based on their inflammatory states^47^. Thus, we generated a single-cell transcriptome reference for explicit M1 and M2 cells, which would allow us to evaluate the inflammatory or anti-inflammatory status of TAMs in a systemic manner. By establishing a reference transcriptome, we confirmed that TAMs in the malignant ascites of GC reflected the features of M2-type macrophages.

In GC patients with ascites, blocking Rho-GTPases RhoA (RhoA) can be an applicable strategy to overcome the poor prognosis conferred by M2-like macrophages. RhoA regulates the migration and invasion of cancer cells induced by M2-like macrophages, and these effects can be attenuated by Rho-associated protein kinase inhibitors^48^. Intriguingly, *RHOA* mutations were enriched in the GS subtype of TCGA^1^. In preclinical studies, RhoA inhibition successfully overcame chemotherapy resistance in both diffuse-type GC stem-like cell models and diffuse GC xenograft models^49^. More research is needed to investigate the effectiveness of RhoA inhibitors in GC, and the modulation of the M2-like phenotype of TAMs by RhoA inhibitors is a potential strategy targeting PC in GC.

Remarkably, we also identified that macrophages collected from metastatic fluid of GC showed the most M2-like features compared to macrophages from other cancer types, such as breast cancer and colorectal cancer (Fig. 4). The reason for the distinct M2-like characteristics in GC ascites is unclear. It is possible that GC has unique tumor characteristics or that they are in the most malignant state, and tumor progression in other cancer types may eventually induce a strong M2 phenotype. To investigate these possibilities, a large-scale study using various cancer types at diverse stages is needed. Moreover, expanding the scope of research to multiomics would reveal the precise tumor-associated mechanisms of M2-like TAMs in various conditions.

In GC, a large number of TAMs could be isolated from late-stage specimens with peritoneal dissemination, which express *CD163* and *CD204*^50^. Late-stage TAMs expressed higher mRNA levels of the anti-inflammatory cytokine IL-10 but lower levels of the pro-inflammatory cytokine TNF-alpha than do early-stage GC TAMs. Importantly, the pro-inflammatory or anti-inflammatory state of macrophages could be converted by different in vitro culture conditions^51^, suggesting that reciprocal M2 to M1 conversion may be achieved in vivo by altering the tumor microenvironment.

After constructing the M2-specific signature gene set from single-cell reference transcriptomes, the prognostic power of the M2 signature was evaluated. In addition, we found that *CITED2* alone has prognostic value in predicting overall survival in GC by representing macrophage M2 properties (Supplementary Fig. 8). CITED2 is a negative regulator of proinflammatory macrophages and promotes anti-inflammatory functions^52^. Depletion of *CITED2* in myeloid cells inhibits PPARγ activation and induces HIF1α stabilization, leading to a proinflammatory response. Our results support the notion that myeloid-specific depletion of *CITED2* could be used as a new immunotherapeutic strategy.

## Supplementary information

Supplementary_information

## Data Availability

All raw sequencing data and the filtered, processed data generated in this study have been deposited in the Gene Expression Omnibus repository under the accession number GSE140182 (private access token: wlofwecmprwtfst). The accession numbers of public datasets are as follows: breast cancer^16^ (GSE75688), colorectal cancer^17^ (EGAS00001001945), primary gastric cancer^19^ (phs001818), antral mucosa biopsies^20^ (GSE134520), and ovarian cancer ascites^21^ (GSE146026).
