# Supplementary material for: Tumor-promoting macrophages prevail in malignant ascites of advanced gastric cancer
Source: Exp Mol Med. 2020 Dec 4;52(12):1976–88. doi: 10.1038/s12276-020-00538-y (PMC8080575; doi:10.1038/s12276-020-00538-y)
Supplement: Supplementary file 1 — Supplementary_information [file 12276_2020_538_MOESM1_ESM.pdf]

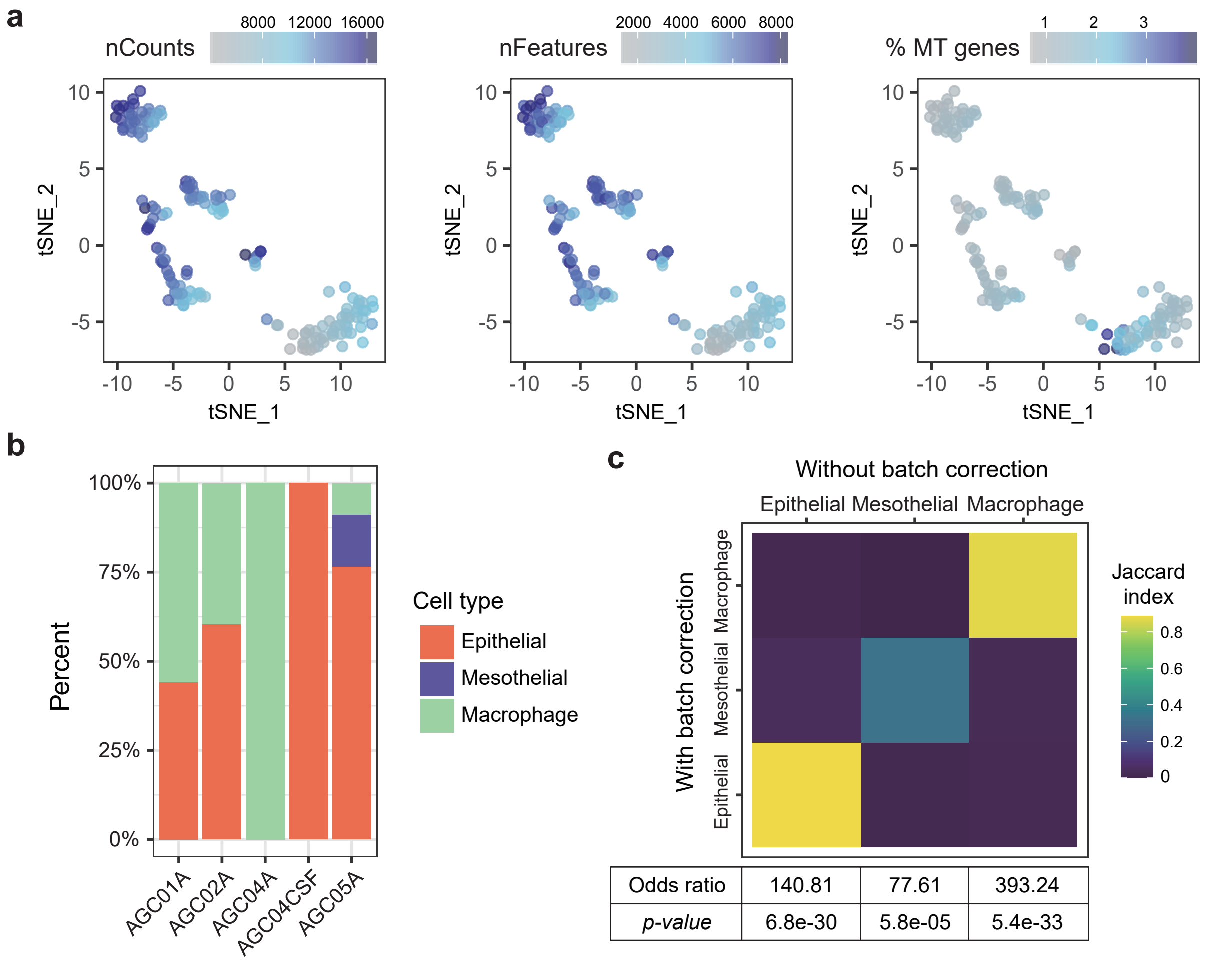

Odds ratio	140.81	77.61	393.24
p-value	6.8e-30	5.8e-05	5.4e-33

Supplementary Fig. 1. Identification of tumor-associated macrophages and cancer cells. a t-distributed stochastic neighbor embedding plots colored by nCounts, nFeatures, or percent of mitochondrial genes show no quality difference among samples. **b** Bar plot of cell type proportion in each sample. **c** Comparison of identified cell types with or without harmony batch correction. 2-D plot (upper) was colored by jaccard index. Odds ratio and *p-value* (lower) were calculated by Fisher's exact test.

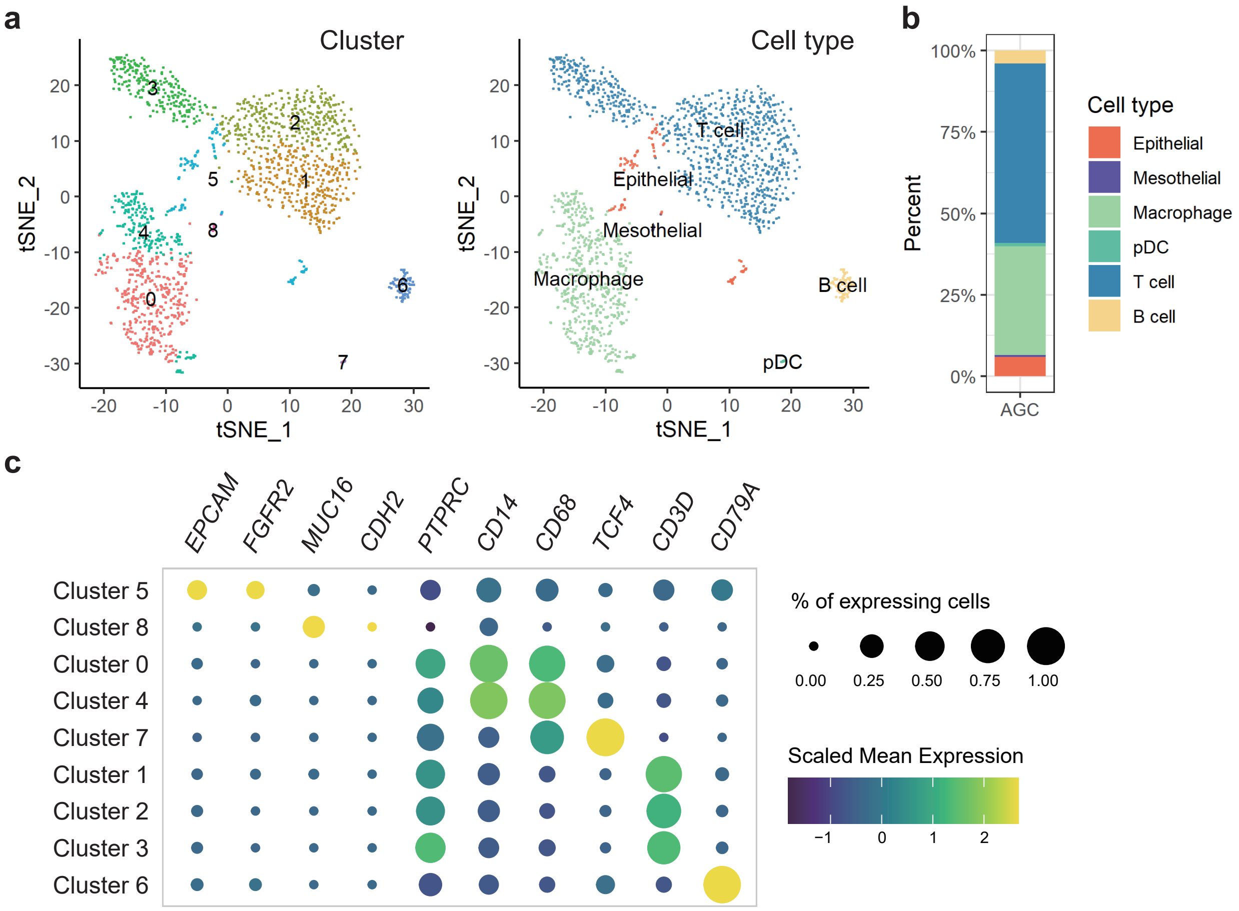

Supplementary Fig. 2. Identification of cell types in malignant ascites of AGC04 (AGC04A) using massively parallel droplet-based method. a t-distribute stochastic neighbor embedding plots colored by SNN cluster (left) or cell type (right). **b** Marker gene expression for epithelial cells, peritoneal mesothelial cells, mesenchymal cells, and macrophages in each cluster.

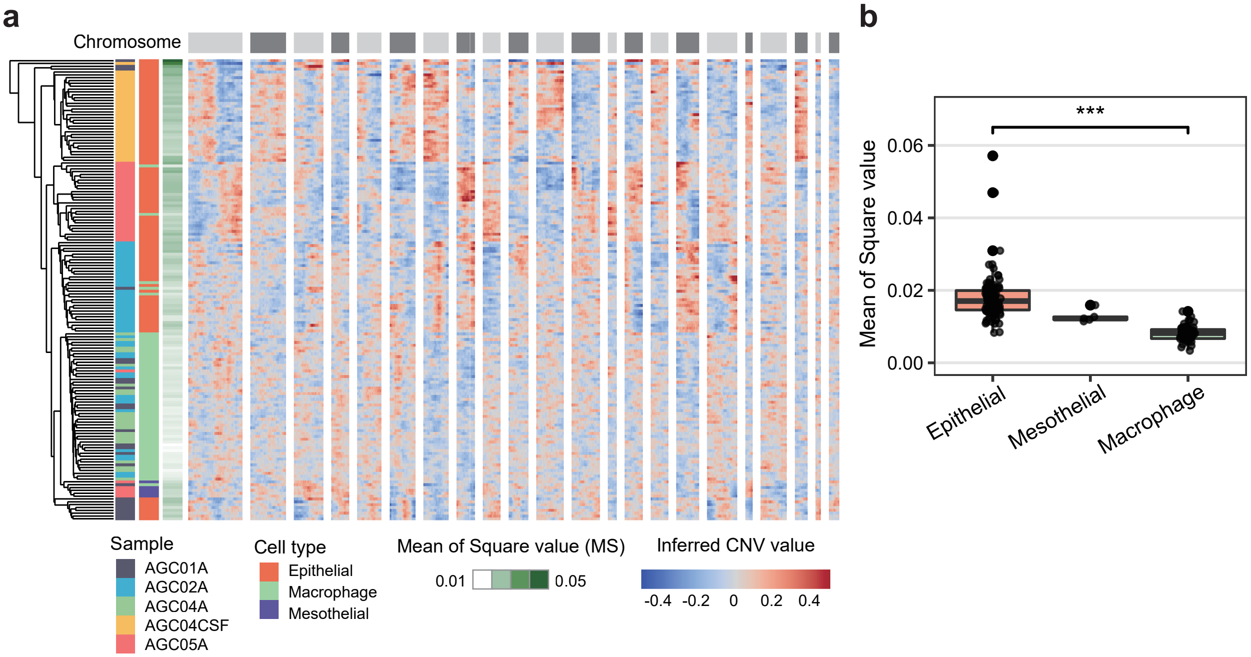

Supplementary Fig. 3. Inferred chromosomal expression patterns of GC single cells. a The chromosomal gene expression patterns (CEP) estimated from single-cell RNA-seq data. Each row represents single cells. Sample origin and defined cell type are annotated with colors on the left side. **b** Epithelial cells show higher variation in chromosomal expression patterns than mesothelial cells or macrophages. Star (***) indicates significant difference (t-test *p-value* < 0.001) between two type of cells.

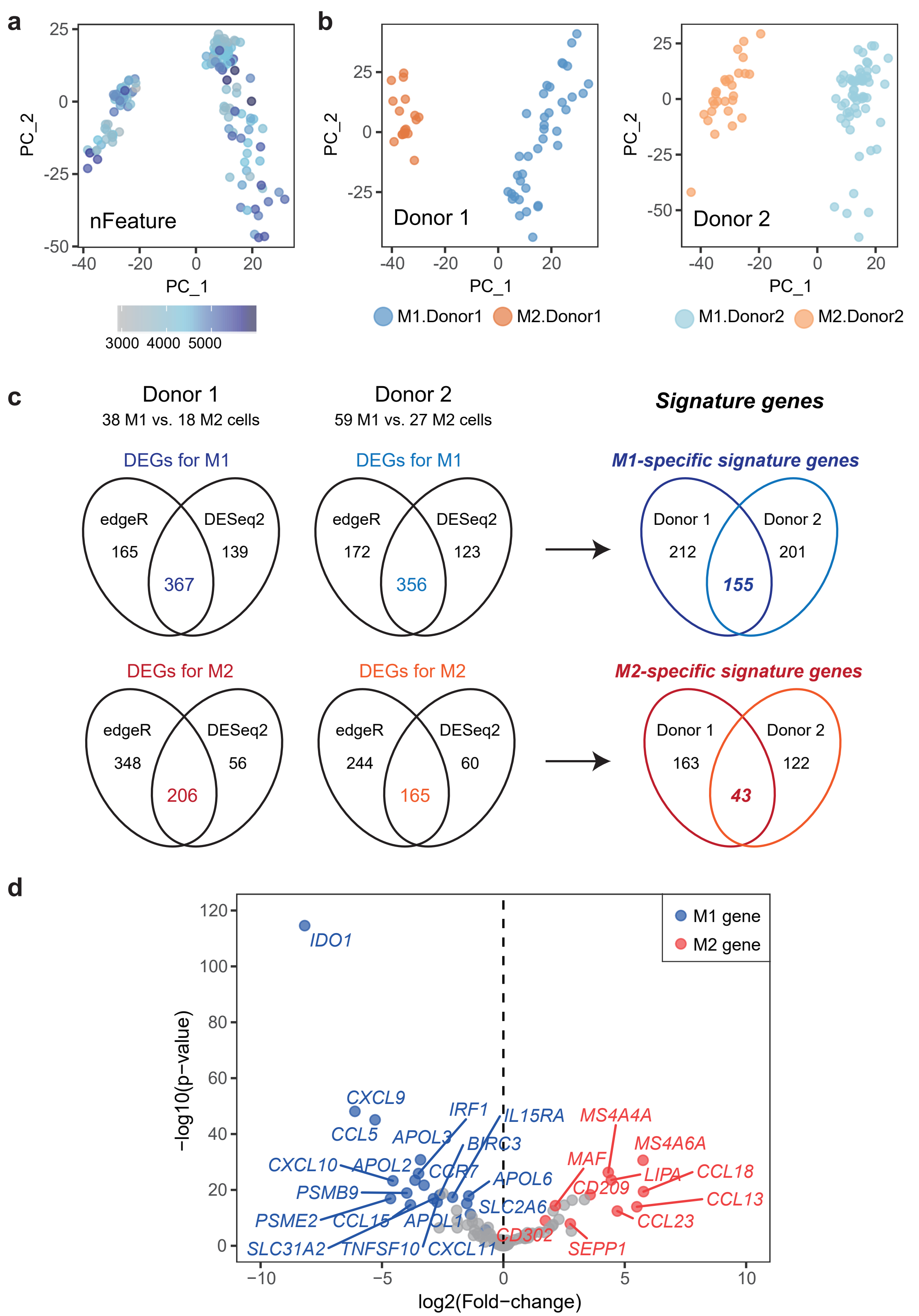

a
nFeature
PC_1
PC_2
3000 4000 5000
b
Donor 1
Donor 2
M1.Donor1
M2.Donor1
M1.Donor2
M2.Donor2
c
Donor 1
38 M1 vs. 18 M2 cells
Donor 2
59 M1 vs. 27 M2 cells
Signature genes
DEGs for M1
edgeR
DESeq2
165
139
367
172
123
356
M1-specific signature genes
Donor 1
Donor 2
212
201
155
DEGs for M2
348
56
206
244
60
165
M2-specific signature genes
163
122
43
d
M1 gene
M2 gene
−log10(p−value)
log2(Fold−change)
IDO1
CXCL9
CCL5
APOL3
IRF1
IL15RA
BIRC3
CXCL10
APOL2
CCR7
APOL6
PSMB9
SLC2A6
PSME2
CCL15
APOL1
SLC31A2
TNFSF10
CXCL11
MS4A4A
MS4A6A
MAF
LIPA
CCL18
CD209
CCL13
CCL23
CD302
SEPP1

Supplementary Fig. 4. Extraction of single-cell transcriptome derived M1- and M2-signature. a tSNE plot colored by nFeatures shows no quality difference among samples and clusters. **b** tSNE plots of cells from each donor. **c** Strategy for extracting M1- and M2-signature genes from two donors using two algorithms (edgeR and DESeq2). **d** The 29 validated genes of the published gene set show a significant difference in expression between M1 and M2 single-cells (*p-value* < 1e5). The x-axis indicates a log2-fold-change in the average expression of M1 and M2 single-cells and the y-axis is a negative log10 of the p-value obtained by a Student's t-test.

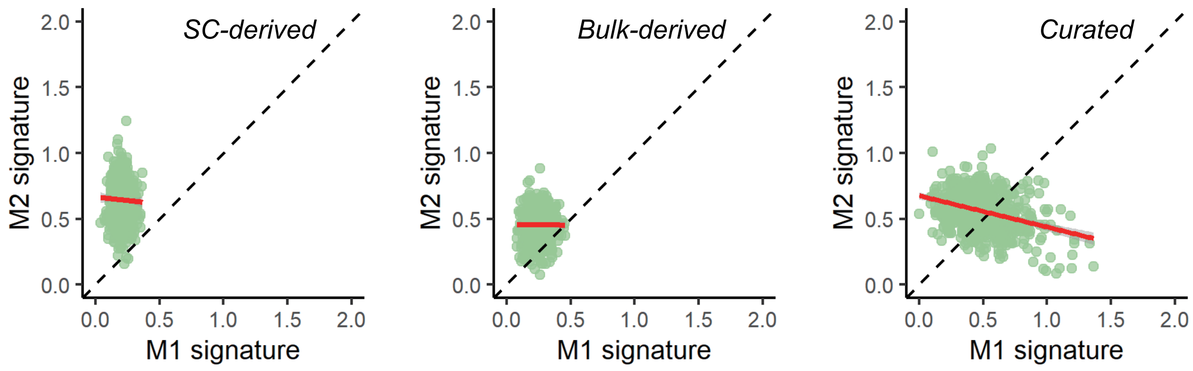

Supplementary Fig. 5. M2-ness of macrophages from malignant ascites of AGC04 (AGC04A) using massively parallel droplet-based method. Two-dimensional dot plots for M1-M2 signature evaluation using three different gene sets; single-cell transcriptome-derived signatures (left), bulk transcriptome-derived signatures (middle), and curated signatures (right). The x-axis for M1-signature and y-axis for M2-signature.

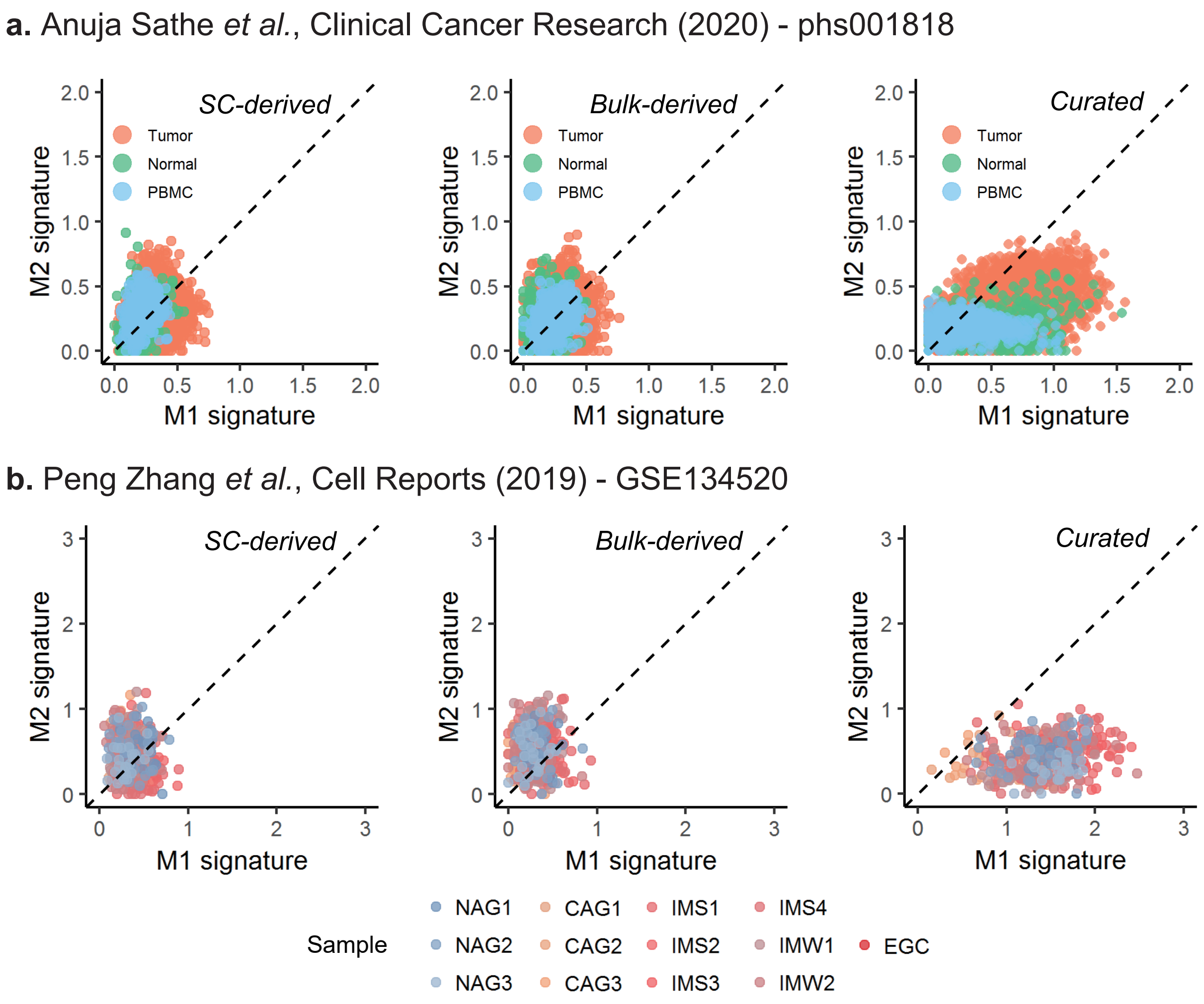

Supplementary Fig. 6. M1-M2 signature of macrophages in stomach tissues. a M1-M2 signature 2-D plots of TAM in gastric cancer tissues shows modest of M2-ness. Public single cell RNA-seq data for gastric cancer tissues, matched normal tissues, and matched PBMC data[19] were analyzed. Each cell was color annotated with their origin of sample. **b** M1-M2 signature 2-D plot of macrophages in non-malignant gastric tissues (NAG, CAG, IM) or early gastric cancer[20]. Each cell was color annotated by sample. NAG, non-atrophic gastritis; CAG, chronic atrophic gastritis; IM, intestinal metaplasia; EGC, early gastric cancer. M1-M2 signature were estimated using three different gene sets; single-cell transcriptome-derived signatures (left), bulk transcriptome-derived signatures (middle), and curated signatures (right).

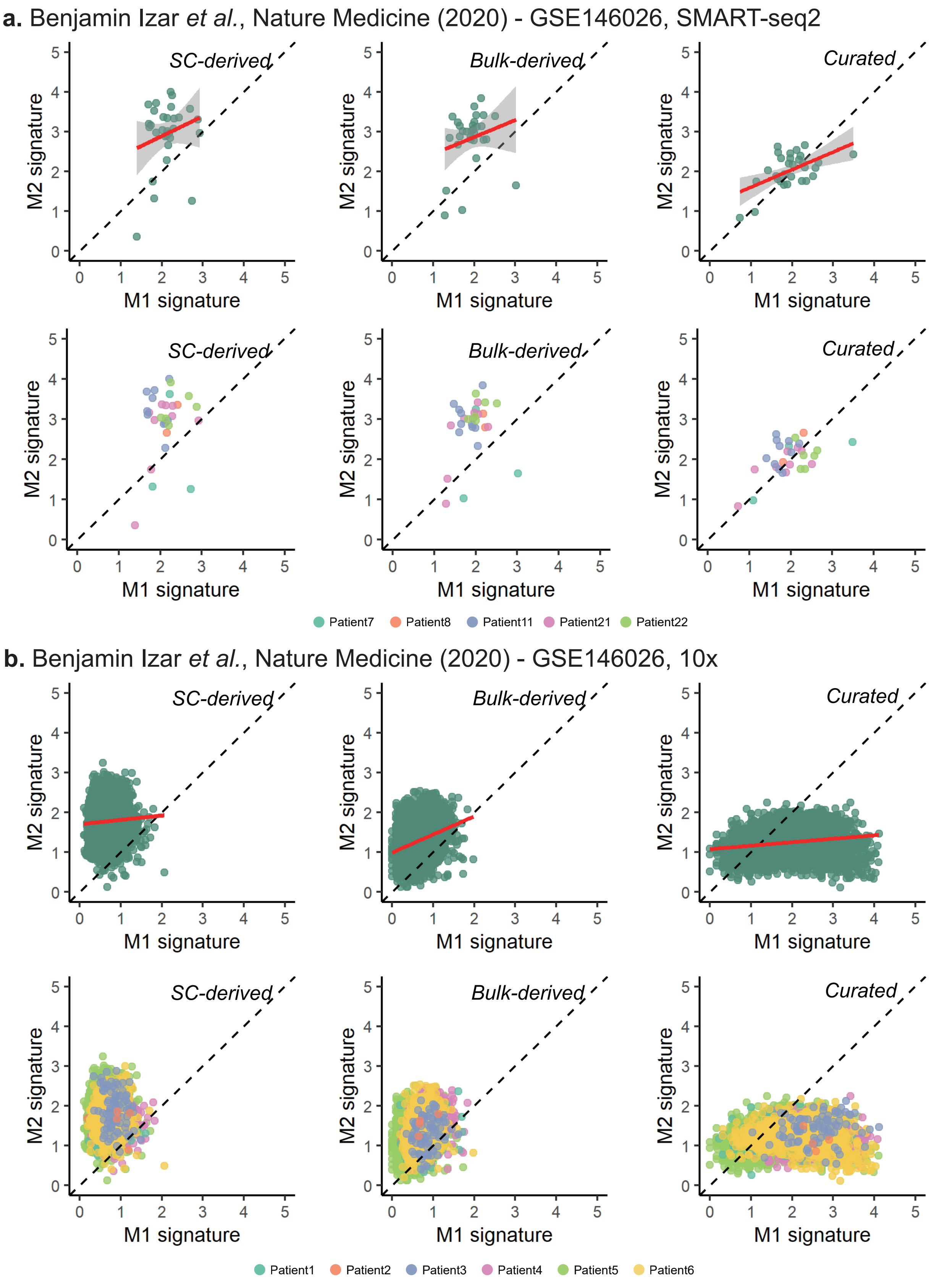
a. Benjamin Izar et al., Nature Medicine (2020) - GSE146026, SMART-seq2
SC-derived
Bulk-derived
Curated
M1 signature
M2 signature
Patient7
Patient8
Patient11
Patient21
Patient22
b. Benjamin Izar et al., Nature Medicine (2020) - GSE146026, 10x
SC-derived
Bulk-derived
Curated
M1 signature
M2 signature
Patient1
Patient2
Patient3
Patient4
Patient5
Patient6

Supplementary Fig. 7. M2-ness of TAM in malignant ascites of ovarian cancer. a M1-M2 signature 2-D plots of TAM in malignant ascites of ovarian cancer [21] using SMART-seq2 data. M1-M2 signature were estimated using three different gene sets; single-cell transcriptome-derived signatures (left), bulk transcriptome-derived signatures (middle), and curated signatures (right). The red line represents the simple linear regression line, and the gray area represents the confidence interval (top). Each cell in the bottom panel is color annotated with a sample. **b** M1-M2 signature 2-D plots of TAM in malignant ascites of ovarian cancer using 10x data.

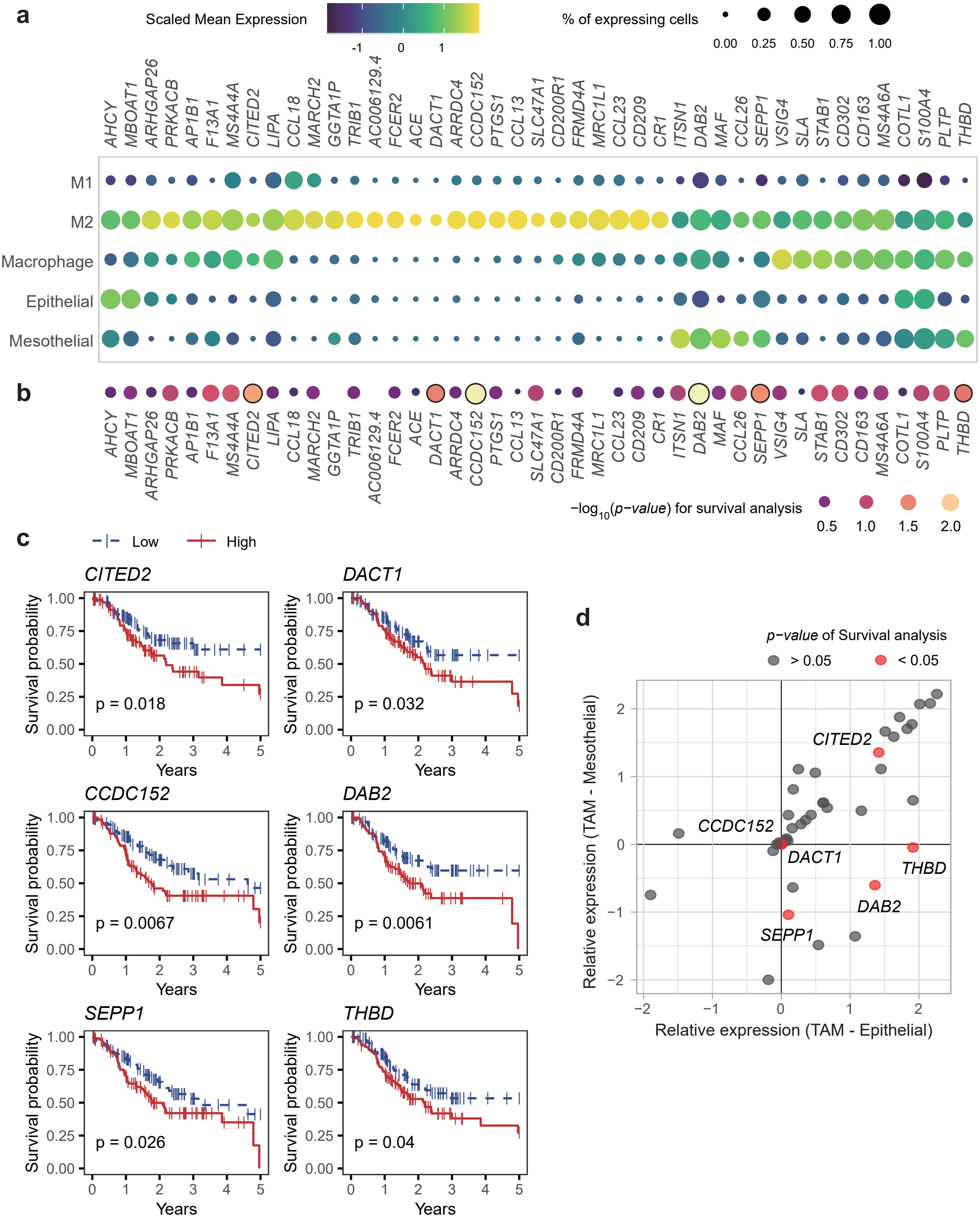

a
Scaled Mean Expression
-1
0
1
% of expressing cells
0.00
0.25
0.50
0.75
1.00
AHCY
MBOAT1
ARHGAP26
PRKACB
AP1B1
F13A1
MS4A4A
CITED2
LIPA
CCL18
MARCH2
GGTA1P
TRIB1
AC006129.4
FCER2
ACE
DACT1
ARRDC4
CCDC152
PTGS1
CCL13
SLC47A1
CD200R1
FRMD4A
MRC1L1
CCL23
CD209
CR1
ITSN1
DAB2
MAF
CCL26
SEPP1
VSIG4
SLA
STAB1
CD302
CD163
MS4A6A
COTL1
S100A4
PLTP
THBD
M1
M2
Macrophage
Epithelial
Mesothelial
b
−log10(p−value) for survival analysis
0.5
1.0
1.5
2.0
c
Low
High
CITED2
p = 0.018
DACT1
p = 0.032
CCDC152
p = 0.0067
DAB2
p = 0.0061
SEPP1
p = 0.026
THBD
p = 0.04
Survival probability
Years
d
p−value of Survival analysis
> 0.05
< 0.05
Relative expression (TAM - Mesothelial)
Relative expression (TAM - Epithelial)
CITED2
CCDC152
DACT1
THBD
DAB2
SEPP1

Supplementary Fig. 8. Prediction of survival rate in TCGA STAD using the M2-signature genes. a Bubble plot for 43 genes of single-cell derived M2-signature per cell type. M1, reference M1; M2, reference M2; Macrophage, TAM of GC; Epithelial, epithelial cancer cells of GC; Mesothelial, mesothelial cells of GC. **b** Overall survival prediction of the single-cell derived M2-signature genes using the TCGA STAD dataset (n = 378). After Kaplan-Meier analysis, significance was represented by a negative log10 of p-value obtained through a log-rank test. Six genes with significance (*p-value* < 0.05) were marked with black circle. **c** Survival plot of six genes which show significant difference in Kaplan-Meier analysis. p indicates p-value of log-rank test. **d** Relative expression of 43 genes in TAM compared to epithelial or mesothelial cells. The x-axis or y-axis indicate the relative expression levels in TAM compared to epithelial or mesothelial cells, respectively.

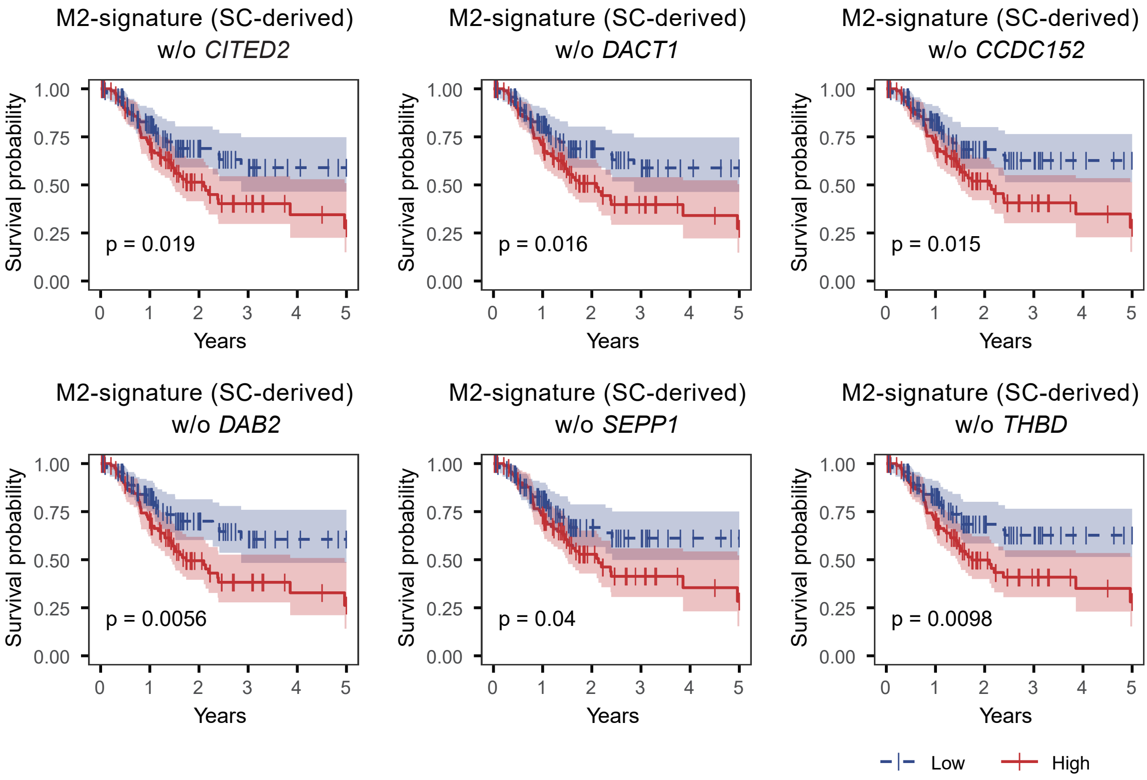

Supplementary Fig. 9. Robustness of survival predictive power using the M2-signature. Single-cell derived M2 signature genes, except *CITED2, DACT1, CCDC152, DAB2, SEPP1*, or *THBD*, still have significance in predicting survival. p indicates p-value of log-rank test.

Supplementary Table 1. Ligand-receptor pair collections

a. Chemokine-related

Pair.Name	Ligand	Receptor	Annotation	Reference	Pair.Source
CSF1_CSF1R	CSF1	CSF1R	Growth Factor	PMID 24202395	PMID 26198319
EGF_CAV1	EGF	CAV1	Growth Factor	PMID 24202395	PMID 26198319
EGF_EGFR	EGF	EGFR	Growth Factor	PMID 24202395	PMID 26198319
EGF_ERBB2	EGF	ERBB2	Growth Factor	PMID 24202395	PMID 26198319
EGF_ERBB3	EGF	ERBB3	Growth Factor	PMID 24202395	PMID 26198319
EGF_ERBB4	EGF	ERBB4	Growth Factor	PMID 24202395	PMID 26198319
EGF_FSHR	EGF	FSHR	Growth Factor	PMID 24202395	PMID 26198319
TGFB1_ACVRL1	TGFB1	ACVRL1	Growth Factor	PMID 24202395	PMID 26198319
TGFB1_CAV1	TGFB1	CAV1	Growth Factor	PMID 24202395	PMID 26198319
TGFB1_CD109	TGFB1	CD109	Growth Factor	PMID 24202395	PMID 26198319
TGFB1_CXCR4	TGFB1	CXCR4	Growth Factor	PMID 24202395	PMID 26198319
TGFB1_ENG	TGFB1	ENG	Growth Factor	PMID 24202395	PMID 26198319
TGFB1_ITGAV	TGFB1	ITGAV	Growth Factor	PMID 24202395	PMID 26198319
TGFB1_ITGB6	TGFB1	ITGB6	Growth Factor	PMID 24202395	PMID 26198319
TGFB1_ITGB8	TGFB1	ITGB8	Growth Factor	PMID 24202395	PMID 26198319
TGFB1_SDC2	TGFB1	SDC2	Growth Factor	PMID 24202395	PMID 26198319
TGFB1_TGFBR1	TGFB1	TGFBR1	Growth Factor	PMID 24202395	PMID 26198319
TGFB1_TGFBR2	TGFB1	TGFBR2	Growth Factor	PMID 24202395	PMID 26198319
TGFB1_TGFBR3	TGFB1	TGFBR3	Growth Factor	PMID 24202395	PMID 26198319
TGFB2_ACVR1	TGFB2	ACVR1	Growth Factor	PMID 24202395	PMID 26198319
TGFB2_ENG	TGFB2	ENG	Growth Factor	PMID 24202395	PMID 26198319
TGFB2_TGFBR1	TGFB2	TGFBR1	Growth Factor	PMID 24202395	PMID 26198319
TGFB2_TGFBR2	TGFB2	TGFBR2	Growth Factor	PMID 24202395	PMID 26198319
TGFB2_TGFBR3	TGFB2	TGFBR3	Growth Factor	PMID 24202395	PMID 26198319
TGFB3_ACVRL1	TGFB3	ACVRL1	Growth Factor	PMID 24202395	PMID 26198319
TGFB3_ENG	TGFB3	ENG	Growth Factor	PMID 24202395	PMID 26198319
TGFB3_TGFBR1	TGFB3	TGFBR1	Growth Factor	PMID 24202395	PMID 26198319
TGFB3_TGFBR2	TGFB3	TGFBR2	Growth Factor	PMID 24202395	PMID 26198319
TGFB3_TGFBR3	TGFB3	TGFBR3	Growth Factor	PMID 24202395	PMID 26198319
CCL2_CCR2	CCL2	CCR2	Cytokine	PMID 24202395	PMID 26198319
CXCL5_CXCR2	CXCL5	CXCR2	Monocyte recruiting	PMID 24202395	PMID 26198319
TNF_LTBR	TNF	LTBR	Pro-inflammatory cytokine	PMID 24202395	PMID 26198319
TNF_SLC5A11	TNF	SLC5A11	Pro-inflammatory cytokine	PMID 24202395	PMID 26198319
TNF_TNFRSF1A	TNF	TNFRSF1A	Pro-inflammatory cytokine	PMID 24202395	PMID 26198319
TNF_TNFRSF1B	TNF	TNFRSF1B	Pro-inflammatory cytokine	PMID 24202395	PMID 26198319
TNF_TNFRSF21	TNF	TNFRSF21	Pro-inflammatory cytokine	PMID 24202395	PMID 26198319
TNF_TRAF2	TNF	TRAF2	Pro-inflammatory cytokine	PMID 24202395	PMID 26198319
IFNG_IFNGR1	IFNG	IFNGR1	Pro-inflammatory cytokine	Interferon gamma	PMID 26198319
IFNG_IFNGR2	IFNG	IFNGR2	Pro-inflammatory cytokine	Interferon gamma	PMID 26198319
IL1A_IL1R1	IL1A	IL1R1	Pro-inflammatory cytokine	Interleukins	PMID 26198319
IL1A_IL1R2	IL1A	IL1R2	Pro-inflammatory cytokine	Interleukins	PMID 26198319
IL1A_IL1RAP	IL1A	IL1RAP	Pro-inflammatory cytokine	Interleukins	PMID 26198319
IL1B_ADRB2	IL1B	ADRB2	Pro-inflammatory cytokine	Interleukins	PMID 26198319

IL1B_IL1R1	IL1B	IL1R1	Pro-inflammatory cytokine	Interleukins	PMID 26198319
IL1B_IL1R2	IL1B	IL1R2	Pro-inflammatory cytokine	Interleukins	PMID 26198319
IL1B_IL1RAP	IL1B	IL1RAP	Pro-inflammatory cytokine	Interleukins	PMID 26198319
IL6_F3	IL6	F3	Pro-inflammatory cytokine	Interleukins	PMID 26198319
IL6_IL6R	IL6	IL6R	Pro-inflammatory cytokine	Interleukins	PMID 26198319
IL6_IL6ST	IL6	IL6ST	Pro-inflammatory cytokine	Interleukins	PMID 26198319
IL12A_CD28	IL12A	CD28	Pro-inflammatory cytokine	Interleukins	PMID 26198319
IL12A_IL12RB1	IL12A	IL12RB1	Pro-inflammatory cytokine	Interleukins	PMID 26198319
IL12A_IL12RB2	IL12A	IL12RB2	Pro-inflammatory cytokine	Interleukins	PMID 26198319
IL12B_IL12RB1	IL12B	IL12RB1	Pro-inflammatory cytokine	Interleukins	PMID 26198319
IL12B_IL12RB2	IL12B	IL12RB2	Pro-inflammatory cytokine	Interleukins	PMID 26198319
IL12B_IL23R	IL12B	IL23R	Pro-inflammatory cytokine	Interleukins	PMID 26198319
IL4_CD53	IL4	CD53	Anti-inflammatory cytokine	Interleukins	PMID 26198319
IL4_IL13RA1	IL4	IL13RA1	Anti-inflammatory cytokine	Interleukins	PMID 26198319
IL4_IL13RA2	IL4	IL13RA2	Anti-inflammatory cytokine	Interleukins	PMID 26198319
IL4_IL2RG	IL4	IL2RG	Anti-inflammatory cytokine	Interleukins	PMID 26198319
IL4_IL4R	IL4	IL4R	Anti-inflammatory cytokine	Interleukins	PMID 26198319
IL5_CSF2RB	IL5	CSF2RB	Anti-inflammatory cytokine	Interleukins	PMID 26198319
IL5_IL5RA	IL5	IL5RA	Anti-inflammatory cytokine	Interleukins	PMID 26198319
IL9_IL2RG	IL9	IL2RG	Anti-inflammatory cytokine	Interleukins	PMID 26198319
IL9_IL9R	IL9	IL9R	Anti-inflammatory cytokine	Interleukins	PMID 26198319
IL10_IL10RA	IL10	IL10RA	Anti-inflammatory cytokine	Interleukins	PMID 26198319
IL10_IL10RB	IL10	IL10RB	Anti-inflammatory cytokine	Interleukins	PMID 26198319
IL10_SIRPG	IL10	SIRPG	Anti-inflammatory cytokine	Interleukins	PMID 26198319
IL13_IL13RA1	IL13	IL13RA1	Anti-inflammatory cytokine	Interleukins	PMID 26198319
IL13_IL13RA2	IL13	IL13RA2	Anti-inflammatory cytokine	Interleukins	PMID 26198319
IL13_IL2RG	IL13	IL2RG	Anti-inflammatory cytokine	Interleukins	PMID 26198319
IL13_IL4R	IL13	IL4R	Anti-inflammatory cytokine	Interleukins	PMID 26198319
CSF1_CSF1R	CSF1	CSF1R	Monocyte recruiting	PMID 28241846	PMID 28241846
CCL20_CCR6	CCL20	CCR6	Monocyte recruiting	PMID 28241846	PMID 28241846
CXCL12_CXCR4	CXCL12	CXCR4	Monocyte recruiting	PMID 28241846	PMID 28241846
CCL18_PITPNM3	CCL18	PITPNM3	Monocyte recruiting	PMID 28241846	PMID 28241846
CSF1_CSF1R	CSF1	CSF1R	Cytokine	PMID 28241846	PMID 28241846
CCL2_CCR2	CCL2	CCR2	Cytokine	PMID 28241846	PMID 28241846
IL4_IL4R	IL4	IL4R	Macrophage polarization	PMID 28241846	PMID 28241846
IL10_IL10RA	IL10	IL10RA	Macrophage polarization	PMID 28241846	PMID 28241846
IL10_IL10RB	IL10	IL10RB	Macrophage polarization	PMID 28241846	PMID 28241846
CCL2_CCR1	CCL2	CCR1	Macrophage polarization	PMID 28241846	PMID 28241846
CCL2_CCR10	CCL2	CCR10	Macrophage polarization	PMID 28241846	PMID 28241846
CCL2_CCR2	CCL2	CCR2	Macrophage polarization	PMID 28241846	PMID 28241846
CCL2_CCR3	CCL2	CCR3	Macrophage polarization	PMID 28241846	PMID 28241846
CCL2_CCR4	CCL2	CCR4	Macrophage polarization	PMID 28241846	PMID 28241846
CCL2_CCR5	CCL2	CCR5	Macrophage polarization	PMID 28241846	PMID 28241846
CCL3_CCR1	CCL3	CCR1	Macrophage polarization	PMID 28241846	PMID 28241846
CCL3_CCR3	CCL3	CCR3	Macrophage polarization	PMID 28241846	PMID 28241846
CCL3_CCR4	CCL3	CCR4	Macrophage polarization	PMID 28241846	PMID 28241846
CCL3_CCR5	CCL3	CCR5	Macrophage polarization	PMID 28241846	PMID 28241846

CCL14_CCR1	CCL14	CCR1	Macrophage polarization	PMID 28241846	PMID 28241846
CCL14_CCR3	CCL14	CCR3	Macrophage polarization	PMID 28241846	PMID 28241846
CCL14_CCR5	CCL14	CCR5	Macrophage polarization	PMID 28241846	PMID 28241846
TGFB1_TGFBR1	TGFB1	TGFBR1	Metastasis	PMID 28241846	PMID 28241846
TGFB1_TGFBR2	TGFB1	TGFBR2	Metastasis	PMID 28241846	PMID 28241846
TGFB1_TGFBR3	TGFB1	TGFBR3	Metastasis	PMID 28241846	PMID 28241846
TGFB2_TGFBR1	TGFB2	TGFBR1	Metastasis	PMID 28241846	PMID 28241846
TGFB2_TGFBR2	TGFB2	TGFBR2	Metastasis	PMID 28241846	PMID 28241846
TGFB2_TGFBR3	TGFB2	TGFBR3	Metastasis	PMID 28241846	PMID 28241846
TGFB3_TGFBR1	TGFB3	TGFBR1	Metastasis	PMID 28241846	PMID 28241846
TGFB3_TGFBR2	TGFB3	TGFBR2	Metastasis	PMID 28241846	PMID 28241846
TGFB3_TGFBR3	TGFB3	TGFBR3	Metastasis	PMID 28241846	PMID 28241846
CCL18_PITPNM3	CCL18	PITPNM3	Metastasis	PMID 28241846	PMID 28241846

b. Macrophage-related

Ligands	Receptors
CSF1	CSF1R
CCL2	CCR1
CCL3	CCR2
CCL4	CCR5
CCL20	CCR6
CD274	PDCD1
IL4	IL4R
IL10	IL10RA
EGF	EGFR
TGFB1	TGFBR1
TGFB2	TGFBR2
TGFB3	TGFBR3
TNF	ACVRL1
IL1B	ACVR1
IL6	TNFRSF1A
IFNG	TNFRSF1B
	IL1R1
	IL1R2
	IL6R
	IL6ST
	IFNGR1
	IFNGR2

Supplementary Table 2. Differentially expressed gene lists for M1 and M2

geneset	SC-derived_M1	SC-derived_M2	Bulk-derived_M1	Bulk-derived_M2	Curated_M1	Curated_M2
source	new	new	Martinez FO et al. J Immunol (2006)	Martinez FO et al. J Immunol (2006)	Azizi E et al. Cell (2018)	Azizi E et al. Cell (2018)
gene	ADAMDEC1	AC006129.4	CCR7	P2RY13	NOS2	ARG1
	ADORA2A	ACE	IL2RA	LPAR6	IL12A	ARG2
	AIM2	AHCY	IL15RA	TGFBR2	IL12B	IL10
	ALCAM	AP1B1	IL7R	HRH1	FCGR1A	CD32
	ANKRD22	ARHGAP26	CXCL11	TLR5	FCGR2A	CD163
	APOL1	ARRDC4	CCL19	CD302	FCGR3A	FCER2
	APOL2	CCDC152	CXCL10	MSR1	CD80	CD200R1
	APOL3	CCL13	CXCL9	CXCR4	CXCR10	PDCD1LG2
	APOL4	CCL18	TNF	CLEC7A	IL23A	CD274
	APOL6	CCL23	CCL5	P2RY14	CXCL9	MARCO
	ARID5B	CCL26	CCL15	CD209	CXCL10	CSF1R
	B4GALT5	CD163	IL12B	CLEC4F	CXCL11	MRC1
	BIRC3	CD200R1	IL15	MS4A6A	CD86	IL1RN
	BTG1	CD209	TNFSF10	CD36	IL1A	IL1R2
	BTN3A2	CD302	IL6	MS4A4A	IL1B	IL4R
	C15orf48	CITED2	CCL20	MRC1	IL6	CCL4
	C1R	COTL1	NAMPT	IGF1	TNF	CCL13
	C1RL	CR1	TYMP	CCL23	CCL5	CCL20
	C1S	DAB2	BCL2A1	CCL18	IRF5	CCL17
	C5orf15	DACT1	FAS	CCL13	IRF1	CCL18
	CCL15	F13A1	BIRC3	SLCO2B1	CD40	CCL22
	CCL5	FCER2	GADD45G	SLC4A7	IDO1	CCL24
	CCR7	FRMD4A	XAF1	SLC38A6	KYNU	LYVE1
	CD38	GGTA1P	SLC7A5	CTSC	CCR7	VEGFA
	CD82	ITSN1	SLCO5A1	HEXB	HLA-DMA	VEGFB
	CDK14	LIPA	SLC2A6	LIPA	HLA-DMB	VEGFC
	CLEC4E	MAF	SLC31A2	ADK	HLA-DOA	FIGF
	CLIC4	MARCH2	IDO1	HNMT	HLA-DOB	EGF
	CLU	MBOAT1	PLA1A	TPST2	HLA-DPA1	CTSA
	CREBRF	MRC1L1	OASL	CERK	HLA-DPB1	CTSB
	CSF2RB	MS4A4A	CHI3L2	HS3ST2	HLA-DQA1	CTSC
	CTD-2319I12.1	MS4A6A	HSD11B1	LTA4H	HLA-DQA2	CTSD
	CUL1	PLTP	AK3	CA2	HLA-DQB1	TGFB1
	CXCL10	PRKACB	SPHK1	ALOX15	HLA-DQB2	TGFB2
	CXCL11	PTGS1	PFKFB3	HS3ST1	HLA-DQB3	TGFB3
	CXCL9	S100A4	PSME2	TGFBI	HLA-DRA	MMP14
	CYP27B1	SEPP1	PFKP	SEPP1	HLA-DRB1	MMP19
	DAPP1	SLA	PSMB9	CHN2	HLA-DRB5	MMP9
	DUSP5	SLC47A1	PSMA2	FN1		CLEC7A
	EBI3	STAB1	OAS2	FGL2		WNT7b
	EDEM1	THBD	PTX3	GAS7		FASLG
	ENTPD1	TRIB1	VCAN	EGR2		TNFSF12
	EPSTI1	VSIG4	APOL3	MAF		TNFSF8

	EREG		IGFBP4			CD276
	ETV7		APOL1			VTCN1
	FAM26F		PDGFA			MSR1
	FAM49A		EDN1			FN1
	GBP1		APOL2			IRF4
	GBP1P1		INHBA			
	GBP2		APOL6			
	GBP4		HESX1			
	GBP5		IRF1			
	GCH1		ATF3			
	GGH		IRF7			
	GPR157					
	HAPLN3					
	HCP5					
	HIST2H4A					
	HIST2H4B					
	HS3ST3B1					
	IDO1					
	IDO2					
	IFI44L					
	IFIT2					
	IFIT3					
	IFITM1					
	IL15RA					
	IL2RG					
	IL32					
	IL3RA					
	IRF1					
	IRG1					
	ISG20					
	ITGB8					
	ITPRIPL2					
	KYNU					
	LAMP3					
	LATS2					
	LGALS17A					
	LILRB4					
	LPAR6					
	LRRK2					
	LY75					
	LYZ					
	MAGED2					
	MEFV					
	MGST1					
	MMP25					
	MT1E					
	MT1F					

	MT1G						
	MT1H						
	MT1L						
	MT1M						
	MT1X						
	MT2A						
	NBN						
	NCF1						
	NEURL3						
	NFKB1						
	NLRC5						
	NUB1						
	NUPR1						
	PARP14						
	PDE4B						
	PDPN						
	PLA2G7						
	PLAUR						
	PNRC1						
	PPA1						
	PPM1K						
	PSMB8						
	PSMB9						
	PSME2						
	PSME2P2						
	PSTPIP2						
	PTGES						
	RARRES3						
	RHBDF2						
	RP11-1143G9.4						
	RP11-274E7.2						
	RP11-44K6.4						
	RSAD2						
	SCO2						
	SEC22B						
	SELM						
	SERPING1						
	SIPA1L1						
	SLAMF7						
	SLC1A2						
	SLC1A3						
	SLC1A4						
	SLC2A6						
	SLC31A2						
	SLC7A11						
	SNX10						
	SOD2						

	STAT4						
	STX11						
	TAP1						
	TAP2						
	TMEM140						
	TMEM38B						
	TNFAIP3						
	TNFAIP6						
	TNFRSF9						
	TNFSF10						
	TNFSF13B						
	TRIM64EP						
	TXN						
	UBD						
	USP11						
	WARS						
	WTAP						
	XPNPEP1						
